# Multifunctional Liposomes Modulate Purinergic Receptor-Induced Calcium Wave in Cerebral Microvascular Endothelial Cells and Astrocytes: New Insights for Alzheimer’s disease

**DOI:** 10.1007/s12035-021-02299-9

**Published:** 2021-01-29

**Authors:** Greta Forcaia, Beatrice Formicola, Giulia Terribile, Sharon Negri, Dmitry Lim, Gerardo Biella, Francesca Re, Francesco Moccia, Giulio Sancini

**Affiliations:** 1 School of Medicine and Surgery, University of Milano-Bicocca, via Cadore 48, 20900 Monza, MB Italy; 2 Nanomedicine Center, Neuroscience Center, School of Medicine and Surgery, University of Milano-Bicocca, via Cadore 48, 20900 Monza, MB Italy; 3 Department of Biology and Biotechnology “Lazzaro Spallanzani”, University of Pavia, Pavia, Italy; 4 Department of Pharmaceutical Sciences, University of Piemonte Orientale, Via Bovio, 6-28100 Novara, Italy

**Keywords:** Liposomes, Alzheimer’s disease, Purinergic receptors, Intracellular calcium waves

## Abstract

In light of previous results, we assessed whether liposomes functionalized with ApoE-derived peptide (mApoE) and phosphatidic acid (PA) (mApoE-PA-LIP) impacted on intracellular calcium (Ca^2+^) dynamics in cultured human cerebral microvascular endothelial cells (hCMEC/D3), as an in vitro human blood-brain barrier (BBB) model, and in cultured astrocytes. mApoE-PA-LIP pre-treatment actively increased both the duration and the area under the curve (A.U.C) of the ATP-evoked Ca^2+^ waves in cultured hCMEC/D3 cells as well as in cultured astrocytes. mApoE-PA-LIP increased the ATP-evoked intracellular Ca^2+^ waves even under 0 [Ca^2+^]_e_ conditions, thus indicating that the increased intracellular Ca^2+^ response to ATP is mainly due to endogenous Ca^2+^ release. Indeed, when Sarco-Endoplasmic Reticulum Calcium ATPase (SERCA) activity was blocked by cyclopiazonic acid (CPA), the extracellular application of ATP failed to trigger any intracellular Ca^2+^ waves, indicating that metabotropic purinergic receptors (P2Y) are mainly involved in the mApoE-PA-LIP-induced increase of the Ca^2+^ wave triggered by ATP. In conclusion, mApoE-PA-LIP modulate intracellular Ca^2+^ dynamics evoked by ATP when SERCA is active through inositol-1,4,5-trisphosphate-dependent (InsP3) endoplasmic reticulum Ca^2+^ release. Considering that P2Y receptors represent important pharmacological targets to treat cognitive dysfunctions, and that P2Y receptors have neuroprotective effects in neuroinflammatory processes, the enhancement of purinergic signaling provided by mApoE-PA-LIP could counteract Aβ-induced vasoconstriction and reduction in cerebral blood flow (CBF). Our obtained results could give an additional support to promote mApoE-PA-LIP as effective therapeutic tool for Alzheimer’s disease (AD).

## Introduction

Alzheimer’s disease (AD) is a neurodegenerative disorder, characterized by alterations in memory formation and storage [1]. It is a progressive neurodegenerative disease with not fully understood etiology. AD may have a vascular origin according to Zlokovic [2] who provided evidences that the aged brain develops a functional uncoupling at the neurovascular unit (NVU), the composite aggregate of cells (neurons, astrocytes, and endothelial cells) which finely tunes cerebral blood flow (CBF) in response to neuronal activity [2]. In AD, we know that Aβ formation and its subsequent accumulation lead to neuronal injury and loss associated with cognitive decline, thus supporting the so-called amyloid hypothesis. According to Zlokovic’s “two hit vascular hypothesis of AD pathogenesis,” Aβ accumulation in the brain is a second insult (hit 2) that is initiated by vascular dysfunction (hit 1) [2]. NVU dysfunction could be an early event in AD and could provide a potential link between this disorder and cerebral ischemia [3]. AD is associated with changes in cerebrovascular structures and functional magnetic resonance imaging (MRI) studies suggest that alterations in CBF regulation in response to cognitive tasks may be a predictor of risk for developing AD [4].

Astrocytes are homeostatic cells in the central nervous system (CNS) [5] and important components of NVU [2]. At early AD stages, astrocytes undergo astrodegeneration and hypotrophy while at later stages of the disease, some of them turn to hypertrophy and astrogliosis in association with deposition of Aβ plaques [6, 7]. Remodeling of astroglial Ca^2+^ signaling toolkit, including metabotropic purinergic signaling, is thought to play a role in these changes [8, 9]. Upon challenge with Aβ and/or during transition to the state of reactivity, astrocytes show enhanced Ca^2+^ signals and become overloaded with Ca^2+^ both in vitro and in vivo [10–13]. Downstream effects of these processes include activation of Ca^2+^/calmodulin-dependent phosphatase calcineurin which, in association with activated microglial cells, drives Aβ-triggered neuroinflammation and astrocytic functional paralysis which are detrimental for neuronal function and survival [14–16].

Balducci and colleagues [17] conducted an in vivo study to investigate the ability of multifunctional liposomes to target Aβ and interact with aggregates; they cross the blood-brain barrier (BBB) promoting their peripheral clearance. These liposomes were bi-functionalized with mApoE (to enhance crossing of the BBB) and with phosphatidic acid (PA), which is a high affinity ligand for Aβ. These bifunctional liposomes (mApoE-PA-LIP) were able to disaggregate Aβ fibrils in vitro, a property that was not exhibited by liposomes mono-functionalized with either mApoE or PA alone [18].

PA is a potent activator of inositol phosphate production and an important role of PA in cell signaling is the increase of intracellular Ca^2+^ ([Ca^2+^]_i_) [19]. PA could act as a positive modulator in different physiological mechanisms; it locally changes membrane topology and may be a key player in membrane trafficking events, where lipid remodeling is crucial [20]. PA could induce membrane curvature and promote fusion, but it also regulates the activity of different proteins involved in these processes [21, 22]. The heterogeneity of PA pathways leads to further investigate its activity to better understand its pleiotropic action in different physiological processes.

An increase in [Ca^2+^]_i_ plays a crucial role within the NVU [23]. Indeed, astrocytic Ca^2+^ signals may regulate local K^+^ concentration and neuronal excitability [24], glutamate release, synaptic plasticity, and control CBF through the production of multiple vasoactive mediators [25]. Likewise, brain microvascular endothelial cells induce vasodilation by nitric oxide (NO) releasing in response to several neurotransmitters and neuromodulators, such as acetylcholine [26], glutamate [27], and histamine [28]. Astrocytic Ca^2+^ signaling could represent a pathway that locally integrates synaptic inputs and controls the microvasculature [24]. In addition, ATP evokes astrocytic Ca^2+^ signals which are triggered by P2Y receptors and stimulate glutamate release, thereby enhancing synaptic strength and increasing CBF [29]. Furthermore, purinergic signaling stimulates brain microvascular endothelial cells via P2Y receptors to locally increase CBF upon release of mediators that improve vasorelaxation [23], including NO and prostaglandins [25]. Activation of P2Y receptors have neuroprotective effects in neuroinflammatory processes [27]. Enhancing purinergic signaling could counteract Aβ-induced vasoconstriction and reduction in CBF [28]. P2Y receptors thus represent important pharmacological targets to treat cognitive dysfunctions and neuropsychiatric diseases [24].

Here, we investigated mApoE-PA-LIP modulation of intracellular Ca^2+^ dynamics in two main NVU elements, cerebral microvascular endothelial cells and astrocytes. In light of the protective role of the purinergic receptor activation, our obtained results could provide a support to promote mApoE-PA-LIP as putative therapeutic tool for AD treatment [30]

## Material and Methods

### Cell Cultures

#### Endothelial Cells

Human cerebral microvascular endothelial cells (hCMEC/D3) were obtained from the Institute Cochin (INSERM, Paris, France). Cells at passages between 27th and 33rd were grown on tissue culture flasks, covered with 0.1mg/ml rat tail collagen type 1, in EndoGRO- MV complete medium (Merck Millipore) supplemented with 1 ng/ml basic FGF (bFGF) and 1% Penicillin–Streptomycin (Life Technologies). Cells were seeded at a density of 24,000–33,000 cells/cm^2^ in T75 flasks and cultured at 37 °C, 5% CO_2_. For calcium imaging experiments, cells were cultured on type 1 collagen-coated coverslips in Petri dishes (p35) at a density of 18,000–24,000 for each Petri containing three coverslips; confluent hCMEC/D3 monolayers were obtained typically by days 3/4.

#### Astrocytes

Immortalized hippocampal astrocytes (iAstro-WT) were gently provided by Dmitry Lim (Department of Pharmaceutical Sciences, University of Piemonte Orientale, Novara, Italy) [31]. Cells at passages between 16th and 22nd were grown on tissue culture flasks in DMEM complete medium (Euroclone) supplemented with 1% Penicillin–Streptomycin (Life Technologies), 10% fetal bovine serum (FBS—Gibco), and 2 mM glutamine (Euroclone). Cells were seeded at a density of 6000–7000 cells/cm^2^ in T75 flasks and cultured at 37 °C, 5% CO_2_. For calcium imaging experiments, cells were cultured in Petri dishes (p35) at a density of 1800–2000 cells. Confluent WT-iAstro monolayers were obtained typically after 2 days of seeding.

### Preparation and Characterization of mApoE-PA-LIP

mApoE-PA-LIP were composed of sphingomyelin (Sm) and cholesterol (Chol) (Sm/Chol 1:1 molar ratio) mixed with 2.5 mol% of 1,2-distearoyl-sn-glycero-3-phosphoethanolamine-N-[maleimide (polyethylene glycol)-2000] (DSPE-PEG-MAL) and with 5 mol % of phosphatidic acid (PA) (International Patent No. PCT/IT2009/000251 of June 10, 2009) [32]. Briefly, lipids were mixed in chloroform/methanol (2:1, v/v) and dried under a gentle stream of nitrogen followed by a vacuum pump for 3 h to remove traces of organic solvent. The resulting lipid film was rehydrated in physiological salt solution (PSS) (for experiments with endothelial cells) or Kreb’s Ringer Buffer (KRB) (for experiments with astrocytes), vortexed, and then extruded 10 times through a polycarbonate filter (100-nm pore size diameter) under 20 bar nitrogen pressure to obtain mApoE-PA-LIP.

mApoE peptide (CWGLRKLRKRLLR, MW 1698.18 g/mol, Karebay Biochem, Monmouth Junction, NJ, USA) was covalently attached on mApoE-PA-LIP surface by thiol–maleimide coupling, to give a final peptide: mal-PEG-PE molar ratio of 1.2:1, as previously described [32].

mApoE-PA-LIP size and polydispersity index (PDI) were obtained using a ZetaPlus particle sizer (Brookhaven Instruments Corporation, Holtsville, NY, U.S.A.) at 25 °C in H2O by dynamic light scattering (DLS) technique with a 652-nm laser beam. The cell viability of hCMEC/D3 is not affected by mApoE-PA-LIP administration up to 200 μM of total lipids (assessed by MTT assay) [18]. We confirm mApoE-PA-LIP non-toxicity also in vivo [17].

### Cell Treatments

#### hCMEC/D3

hCMEC/D3 were cultured on coverslips, maintained in a low-profile chamber with physiological salt solution (PSS) (NaCl 150 mM; KCl 6 mM; MgCl2 1 mM; CaCl_2_ 1.5 mM; HEPES 10 mM; Glucose 10 mM). Ca^2+^-free solution (0 [Ca2+]_e_) was obtained by substituting Ca^2+^ with 2 mM NaCl and by adding 0.5 mM EGTA.

ATP (50μM) was added to the PSS and 0 [Ca^2+^]_e_ solutions. Cyclopiazonic acid (10 μM) was added to the PSS and 0 [Ca^2+^]_e_ solutions. Then, mApoE-PA-LIP were dissolved at a final concentration of 0.01mg/ml (total lipids) in PSS and 0 [Ca^2+^]_e_ solutions.

#### iAstro-WT

iAstro KRB solution (125mM NaCl, 5mM KCl, 1mM Na_2_HPO_4_, 1mM MgSO_4_, 5.5mM glucose, 20mM HEPES, pH 7.4) was supplemented with 2mM CaCl_2_. ATP (100μM) was added to the solution (both KRB and 0 [Ca^2+^]_e_ KRB). Cyclopiazonic acid (10μM) was added to the PSS solution (both KRB and 0 [Ca^2+^]_e_ KRB). mApoE-PA-LIP were dissolved at a final concentration of 0.01mg/ml (total lipids) in PSS (both KRB and 0 [Ca^2+^]_e_ KRB).

All solutions were titrated to pH 7.4 with NaOH.

### [Ca^2+^]_i_ Measurements

hCMEC/D3 and iAstro-WT were loaded with Fura-2AM (4μM) in PSS for 30 min at 37 °C away from light. The coverslip, after being washed in PSS, was disposed in a low-profile chamber and maintained in physiological solution at 37 °C for the entire duration of the experiments. Fura-2 fluorescence ratio (excitation at 340 and 380 nm; emission at 510 nm) was observed by wide-field fluorescence time lapse Nikon Eclipse FN1 upright microscope (Nikon Corp., Tokyo, Japan) equipped with a 60X Nikon objective (water-immersion, 2.0 mm working distance, 1 numerical aperture). For experiments with astrocytes, we used 40X Nikon objective (water-immersion, 3.5 mm working distance, 0.80 numerical aperture). The excitation filters were mounted on a filter wheel (Lambda 10-2, Sutter Instrument, Novato, CA, USA). The fluorescent signal was collected by means of a Coolsnap Photometrics CCD camera through a bandpass 510-nm filter.

By using MetaFluor (Molecular Devices, Sunnyvale, CA, USA) software, we measured and plotted online, every 1200 ms, the fluorescence from 8–12 regions of interest (ROI) inside each loaded cell; each ROI was identified by a number. Changes in intracellular Ca^2+^ levels monitored by measuring, for each ROI, the ratio (340/380) of the mean fluorescence. For the entire duration of the experiment, ratio measurements were performed and plotted online every 1200 ms with 800 ms exposure time. Duration and area values were measured using Origin tools per each response in different conditions.

### Chemicals

Cholesterol (chol), phosphatidic acid (PA), sphyngomielin, and 1,2-distearoyl-sn-glycero-3- phospho-ethanolamine-N [maleimide(polyethyleneglycol)-2000] (DSPE-PEG-mal) were from Avanti Polar Lipids Inc (Alabaster, AL, USA). Adenosine 5′-triphosphate disodium salt hydrate (ATP) and cyclopiazonic acid (CPA—1 mM stock in dimethyl sulfoxide—DMSO) were obtained from Sigma Aldrich (C1530–5MG).

mApoE peptide (CWGLRKLRKRLLR, MW 1698.18 g/mol) was synthetized by Karebay Biochem (Monmouth Junction, NJ, USA). Fura-2 acetoxymethyl ester (Fura2/AM - 1 mM stock in DMSO) was obtained from Thermo Fisher. This indicator has an emission peak at 505 nm and changes its excitation peak from 340 to 380 nm in response to Ca^2+^ binding.

### Statistics

All data have been collected from hCMEC/D3 and iAstro-WT. The 1st spike amplitude evoked by ATP was measured considering the value from the baseline before and after the stimulus trigger. Area under the curve (A.U.C) was obtained using Origin Integration function. Statistical analysis was performed using Microsoft Office Excel. Pooled data were given as mean ± SE and statistical significance was evaluated by the Student’s *T* test for unpaired observations with Gaussian distributions and by the Mann-Whitney non-parametric test with non-Gaussian distributions. Differences were considered significant at *p value < 0.05, **p value < 0.01, and ***p value < 0.001.

## Results

### mApoE-PA-LIP Synthesis and Characterization

The total lipid recovery for NL after extrusion was about 70%. The different lipid components of the mixtures were recovered with equal efficiency and always reflected the proportion in the starting mixture. The yield of NL coupling with mApoE was 70 ± 9%. Final preparations of mApoE-PA-LIP had a diameter of 122.7 ± 4.85 nm with a PDI of 0.1 ± 0.02.

### Endoplasmic Reticulum Ca^2+^ Release Is Present and Results in Store-Operated Ca^2+^ Entry Activation in hCMEC/D3

**Fig. 1:**
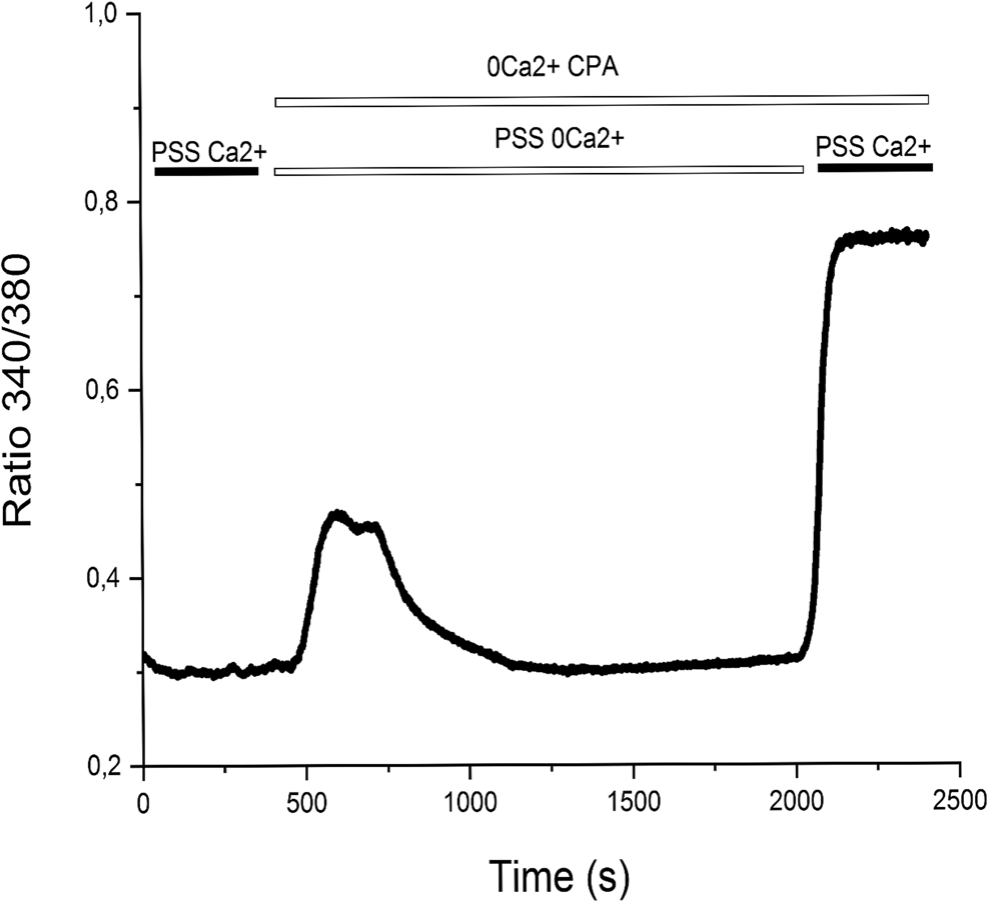
“Add-back” protocol. In the Ca^2+^ “add-back” protocol, CPA (10μM) was administered under 0 [Ca^2+^]_e_ conditions to deplete the ER Ca^2+^ pool and activate store-operated calcium channels, as indicated by the second increase in [Ca^2+^]_i_ arising after restitution of external Ca^2+^

The Ca^2+^ “add-back” protocol is a widely employed protocol to monitor both endogenous Ca^2+^ release and SOCE (store-operated Ca^2+ ^entry) in non-excitable cells [33], including vascular endothelial cells [28, 34]. This protocol consists in incubating the cells with a specific inhibitor of SERCA, such as thapsigargin or cyclopiazonic acid (CPA), under 0 [Ca^2+^]_e_ condition to evaluate passive ER (endoplasmic reticulum) Ca^2+^ egression through leakage channels. Subsequently, restitution of extracellular Ca^2+^ induces a second increase in [Ca^2+^]_i_ which is due to Ca^2+^ entry through open store-operated Ca^2+^ channels. As reported in Fig. 1, CPA (10 μM) elicited a first transient increase in [Ca^2+^]_i_, which reflected the depletion of the ER Ca^2+^ pool, followed by massive SOCE activation arising after extracellular Ca^2+^ restitution. These data are consistent with those recently described in hCMEC/D3 cells [26].

### mApoE-PA-LIP Pre-treatment Increases ATP-Evoked Calcium Waves in hCMEC/D3 Cells

**Fig. 2:**
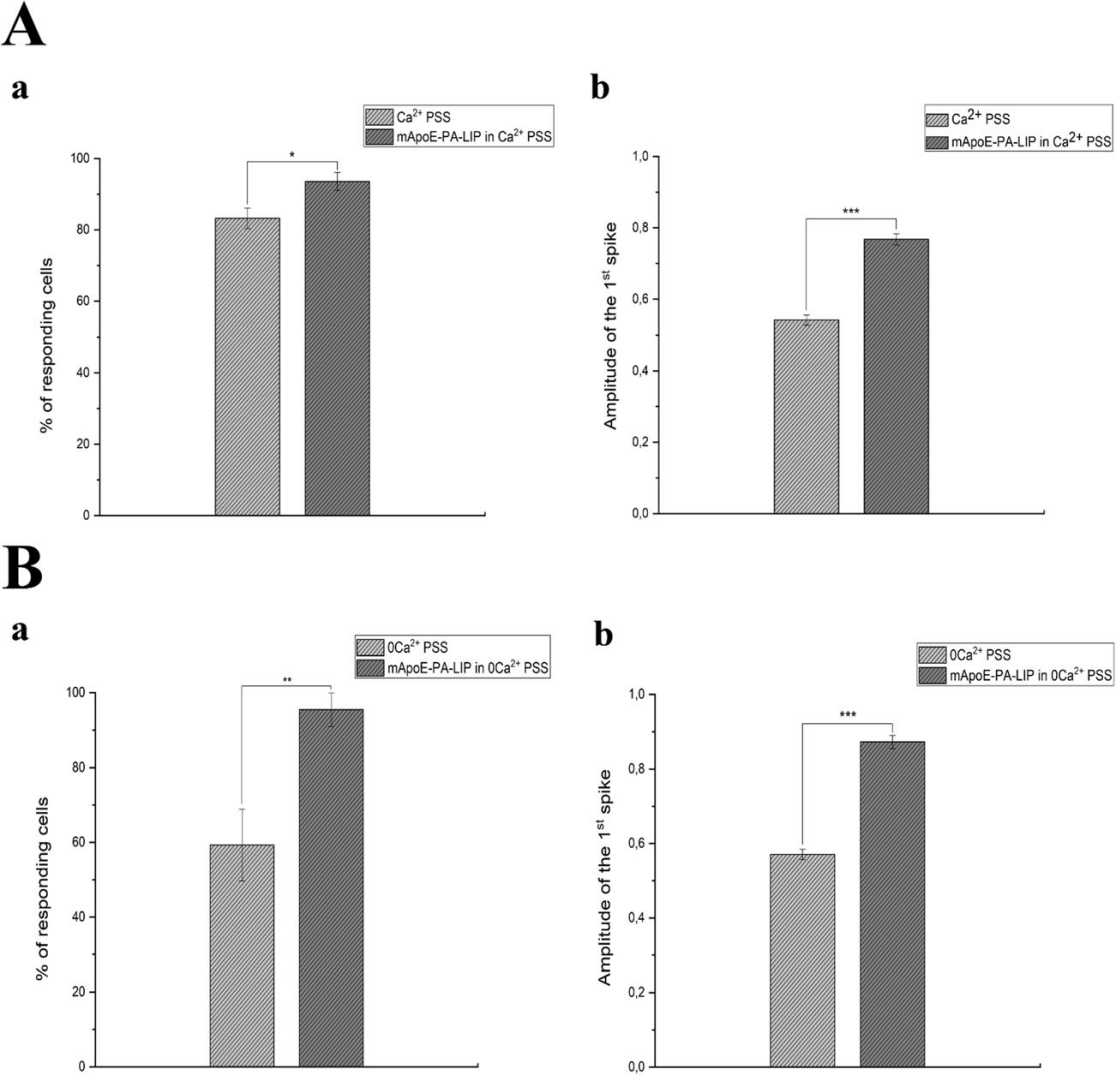
(A-a) bar histogram shows the average ± SE of the percentage of ATP (50 μM) responding cells in PSS compared to the average ± SE of the percentage of ATP (50 μM) responding cells after 5 min pre-treatment with 0.01mg/ml mApoE-PA-LIP (increase of 10.3%). (A-b) Bar histogram shows the average ± SE of the amplitude of the 1st spike under the same conditions of (A-a). (B-a) Bar histogram shows the average ± SE of the percentage of ATP (50 μM) responding cells in 0 [Ca^2+^]_e_ PSS compared to the average ± SE of the percentage of ATP (50 μM) responding cells after 5 min pre-treatment with 0,01mg/ml mApoE-PA-LIP (increase of 36.2%). (B-b) Bar histogram shows the average ± SE of the amplitude of the 1st spike under the same conditions of (B-a). Differences were considered significant at *p value < 0.05, **p value < 0.01, and ***p value < 0.001

A recent investigation revealed that ATP induced an increase in [Ca^2+^]i by stimulating P2Y2 receptors in hCMEC/D3 cells [27]. In standard PSS buffer, a pre-treatment with 0.01mg/ml mApoE-PA-LIP (*n* = 87) of the duration of 5 min increased the Ca^2+^ dynamics evoked by a short (30 sec) ATP pulse in comparison to control conditions (*n* = 139) (Fig. 2-A). In particular, we found that the percentage of responding hCMEC/D3 cells increased by 10.3% (Fig. 2-Aa) in presence of mApoE-PA in standard PSS solution. A pre-treatment with 0.01mg/ml mApoE-PA-LIP increased by 36.2% the percentage of ATP responding cells (*n* = 21) in comparison to controls (*n* = 16) also in the absence of extracellular Ca^2+^ (0 [Ca^2+^]_e_) (Fig. 2-Ba). Furthermore, we observed an increase of the ATP-evoked calcium peak both in PSS buffer and in 0 [Ca^2+^]_e_ (Fig. 2-Ab and 2-Bb). Bar histogram shows the average ± SE of the percentage of ATP responding cells and of the amplitude of the 1st spike.

**Fig. 3:**
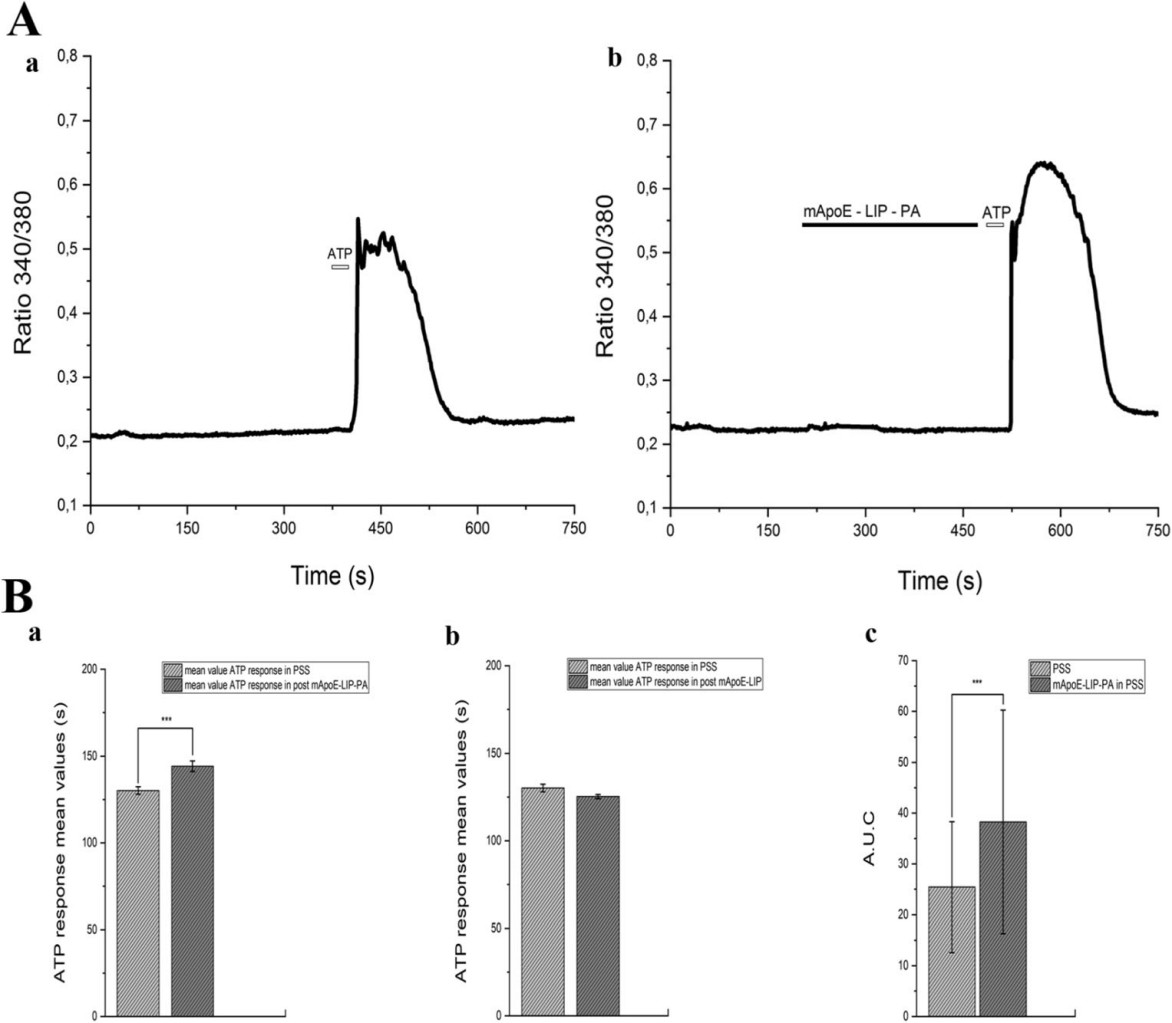
(A-a) hCMEC/D3 ATP (50 μM) response. (A-b) hCMEC/D3 ATP (50 μM) response after pre-treatment with 0.01mg/ml mApoE-PA-LIP in Ca^2+^ PSS. (B-a) Bar histogram of the ATP response mean values ± SE in PSS and after a mApoE-PA-LIP pre-treatment. (B-b) Bar histogram of the ATP response mean values ± SE in PSS and after a mApoE-LIP pre-treatment. (B-c) Bar histogram of the A.U.C mean values ± SE in PSS and after a mApoE-PA-LIP pre- treatment. Differences were considered significant at *p value < 0.05, **p value < 0.01, and ***p value < 0.001

We then analyzed the duration and the A.U.C. of the Ca^2+^ response to ATP in control condition and after 5 min pre-treatment with 0.01mg/ml mApoE-PA-LIP (Fig. 3-A). A significant increase (mean ± SE, 144 ± 3.03 s, *n* = 87) of the duration of the ATP-evoked Ca^2+^ waves was found in presence of mApoE-PA-LIP in comparison to controls (mean ± SE, 130 ± 2.19 sec, *n* = 139) (Fig. 3-Ba). The pre-treatment with mApoE-LIP without PA functionalization did not increase the mean duration of the ATP-induced Ca^2+^ response in hCMEC/D3 cells (mean ± SE, 125 ± 1.95 sec, *n* = 52) (Fig. 3-Bb). In agreement with the elongation of the intracellular Ca^2+^ wave, we observed a significant increase in the A.U.C. value after the mApoE-PA-LIP pre-treatment (mean A.U.C ± SE 38.26 ± 5.06) in comparison to controls (mean A.U.C ± SE 25.44 ± 2.82) (Fig. 3-Bc).

**Fig. 4:**
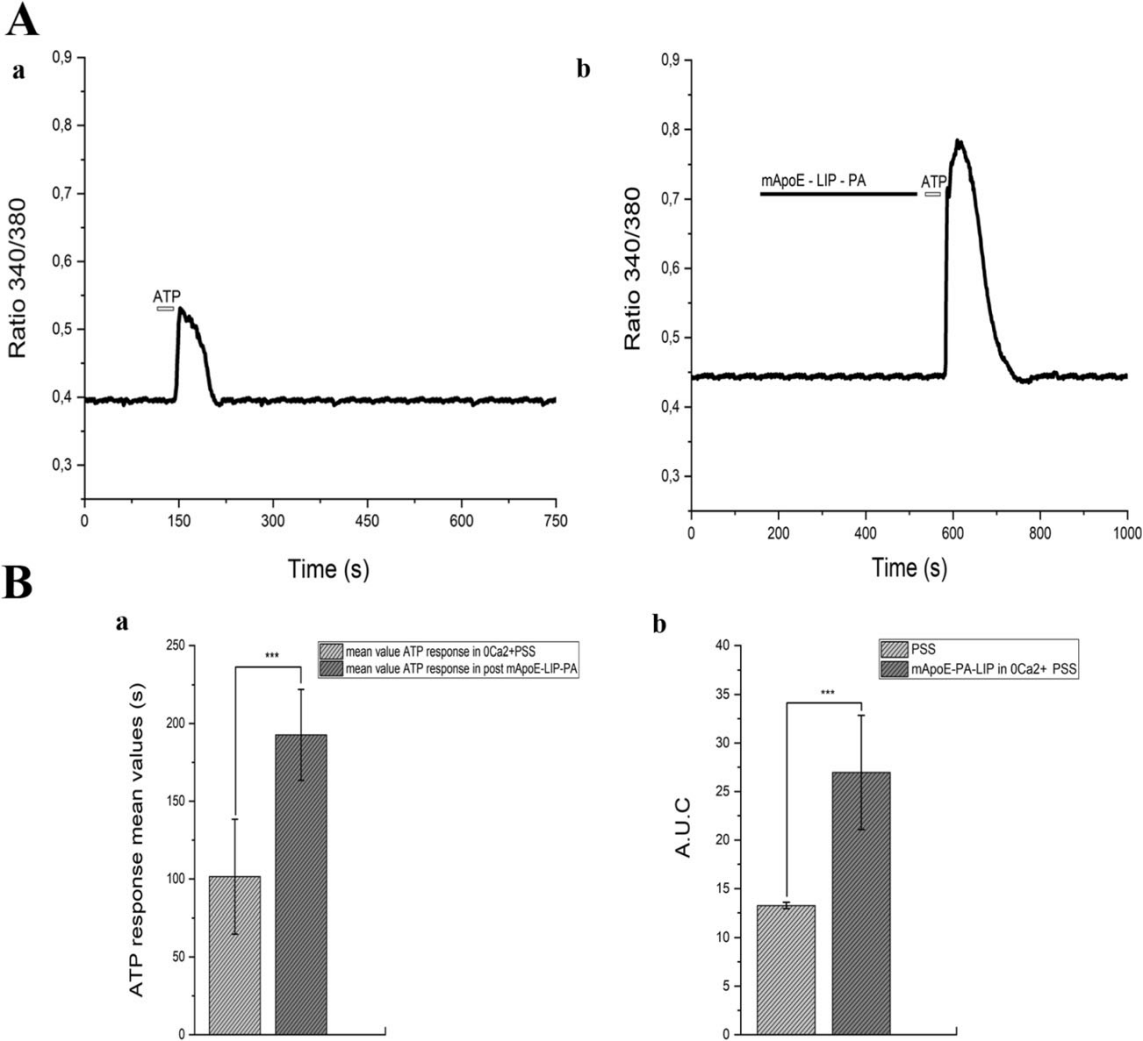
(A-a) hCMEC/D3 ATP (50 μM) response in 0 [Ca^2+^]_e_ PSS. (A-b) hCMEC/D3 ATP (50 μM) response after pre-treatment with 0.01mg/ml mApoE-PA-LIP in 0 [Ca^2+^]_e_ PSS. (B-a) Bar histogram of the ATP response mean values ± SE in PSS and after a mApoE-PA-LIP pre- treatment in 0 [Ca^2+^]_e_ PSS. The asterisk indicated *p* value < 0.05. (B-b) Bar histogram of the A.U.C mean values ± SE in PSS and after a mApoE-PA-LIP pre-treatment. Also, in 0 [Ca^2+^]_e_, the pre-treatment with mApoE-PA-LIP increased the calcium dynamics evoked by ATP stimulus in comparison to control. Differences were considered significant at *p value < 0.05, **p value < 0.01, and ***p value < 0.001

Moreover, we found again that the pre-treatment with mApoE-PA-LIP in the absence of extracellular Ca^2+^ 0 [Ca^2+^]_e_ increased the duration of ATP-evoked Ca^2+^ waves (mean ± SE, 192.7 ± 6.38 sec, *n* = 21) in comparison to controls (mean ± SE, 101.5 ± 9.2 sec, *n* = 16) (Fig. 4-A and 4-Ba). Likewise, also the A.U.C increased (mean A.U.C ± SE 26.97 ± 5.88) in comparison to controls (mean A.U.C ± SE 13.29 ± 0.33) (Fig. 4-Bb).

### Astrocyte Pre-treatment with mApoE-PA-LIP Increases ATP Response and Amplitude

**Fig. 5:**
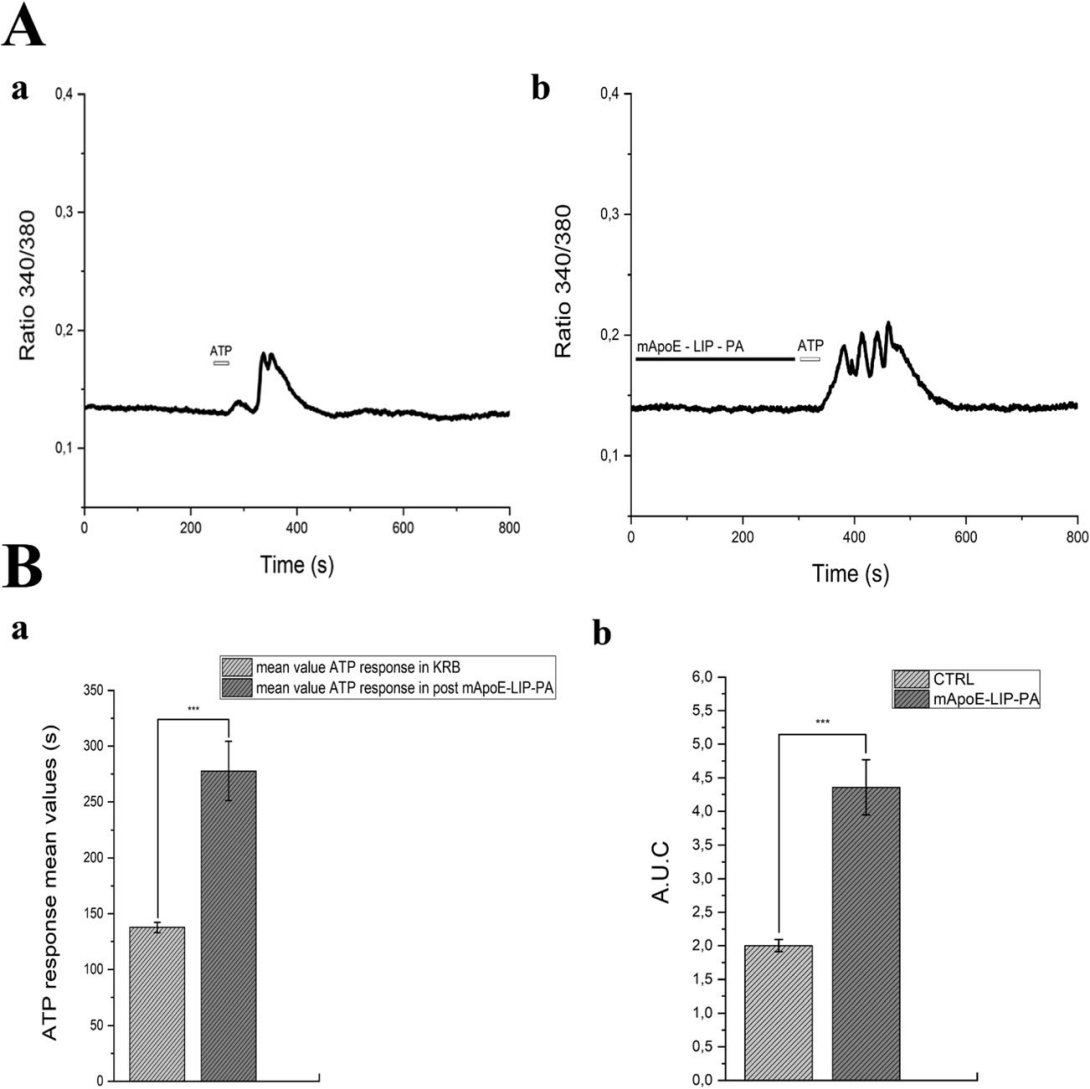
(A-a) iAstro-WT ATP (100μM) response. (A-b) I Astro-WT ATP (100 μM) response after pre-treatment with 0.01 mg/ml mApoE-PA-LIP in Ca^2+^ KRB. (B-a) Bar histogram of the ATP response mean values ± SE in KRB and after mApoE-PA-LIP pre-treatment. (B-b) Bar histogram of the A.U.C mean values ± SE in PSS and after mApoE-PA-LIP pre-treatment. Differences were considered significant at *p value < 0.05, **p value < 0.01, and ***p value < 0.001

We then stimulated iAstro-WT with ATP in control condition and after 10-min pre-incubation with 0.01mg/ml mApoE-PA-LIP (Fig. 5Aa–b). A recent investigation showed that P2Y1 receptors trigger ATP-induced intracellular Ca^2+^ signals in hippocampal astrocytes [34]. A significant increase (mean ± SE, 277 ± 26.63 sec, *n* = 34) in the duration of ATP-evoked evoked Ca^2+^ waves was evident in presence of mApoE-PA-LIP in comparison to controls (mean ± SE, 137 ± 4.65 sec, *n* = 56, *p* value < 0.001) (Fig. 5B-a).

**Fig. 6:**
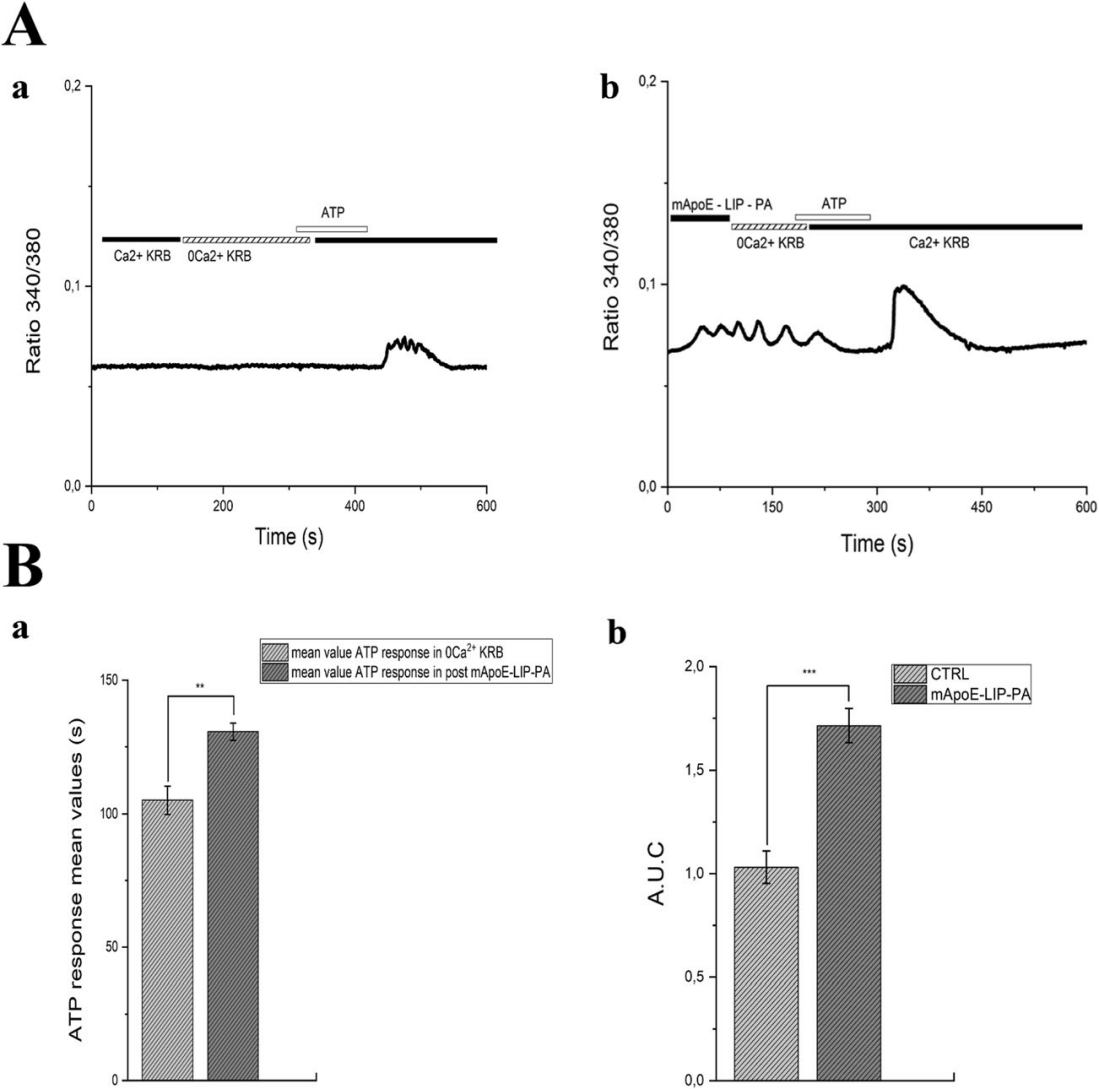
(A-a) iAstro-WT ATP (100 μM) response in 0 [Ca^2+^]_e_ KRB. (A-b) iAstro-WT ATP (100 μM) response after pre-treatment with 0.01 mg/ml mApoE-PA-LIP in 0 [Ca^2+^]_e_ KRB; (B-a) Bar histogram of the ATP response mean values ± SE and after mApoE-PA-LIP pre-treatment in 0 [Ca^2+^]_e_ KRB. The asterisk indicates *p* value < 0.05. (B-b) Bar histogram of the A.U.C mean values ± SE and after mApoE-PA-LIP pre-treatment in 0 [Ca^2+^]_e_ KRB. In 0 [Ca^2+^]_e_, the pre-treatment with mApoE-PA-LIP increased the calcium dynamics evoked by ATP stimulus in comparison to control. Differences were considered significant at *p value < 0.05, **p value < 0.01, and ***p value < 0.001

We then confirmed that also the A.U.C. (Fig. 5B-b) of the Ca^2+^ response to ATP increased after pre-treatment with mApoE-PA-LIP in PSS. After the pre-treatment, indeed, A.U.C increased (4.35 ± 0.41 s) in comparison to control (2 ± 0.09 s). We confirmed that the pre-treatment with mApoE-PA-LIP in absence of extracellular Ca^2+^ (0 [Ca^2+^]_e_) increased ATP-evoked Ca^2+^ waves in comparison to control (Fig. 6Aa–b). The ATP response duration (Fig. 6B-a) was significantly increased (mean ± SE, 130.68 ± 3.25 sec, *n* = 21) in comparison to control (mean ± SE, 102.47 ± 5.98 sec, *n* = 38). Under this condition, also the A.U.C value increased (1.71 ± 0.07) in comparison to control (A.U.C ± SE 1± 0.08) (Fig. 6B-b).

### mApoE-PA-LIP Pre-treatment Modulates Ca^2+^ Dynamics when SERCA Is Active Both in hCMEC/D3 Cells and iAstro-WT

**Fig. 7:**
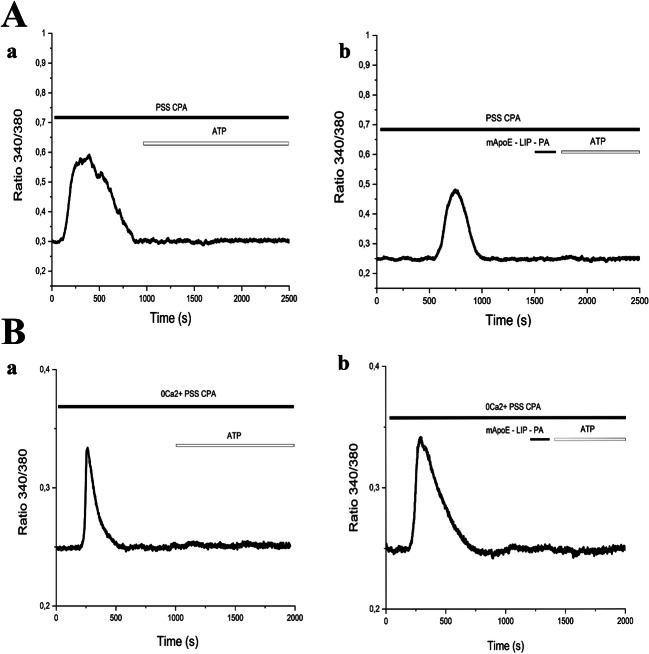
(A-a) CPA (10μM) response under extracellular Ca^2+^ conditions. ATP-evoked response is blocked by CPA perfusion. (A-b) CPA (10μM) response after pre-treatment with 0.01 mg/ml mApoE-PA-LIP, also in these conditions there is no ATP response. (B-a) CPA (10μM) response under 0 [Ca^2+^]_e_ conditions. (B-b) CPA (10μM) response after pre-treatment with 0.01 mg/ml mApoE-PA-LIP in 0 [Ca^2+^]_e_ PSS. In presence of CPA both in presence of extracellular calcium and in 0 [Ca^2+^]_e_ ATP failed to activate calcium wave

**Fig. 8:**
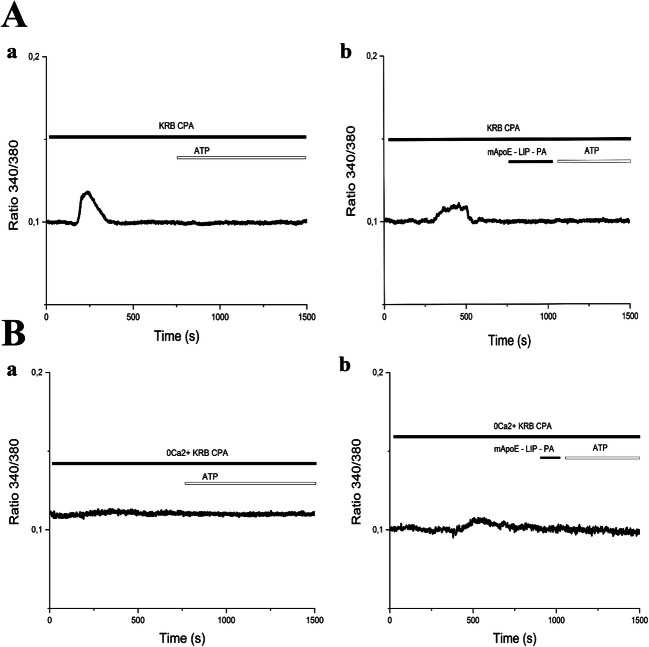
(A-a) CPA (10μM) response under extracellular Ca^2+^ conditions. ATP-evoked response is blocked by CPA perfusion. (A-b) CPA (10 μM) response after pre-treatment with 0.01 mg/ml mApoE-PA-LIP, also in these conditions there is no ATP response. (B-a) CPA (10μM) administration under 0 [Ca^2+^]_e_ conditions. (B-b) CPA (10μM) response after pre-treatment with 0.01 mg/ml mApoE-PA-LIP in 0 [Ca^2+^]_e_ KRB. In presence of CPA both under calcium and in 0 [Ca^2+^]_e_ ATP failed to activate calcium wave

P2Y1 and P2Y2 receptors were shown to elicit, respectively, astrocytic and endothelial Ca^2+^ waves by stimulating phospholipase Cβ (PLCβ), thereby inducing InsP3-dependent Ca^2+^ release from the ER. In order to confirm that the ER represents the main endogenous Ca^2+^ store targeted by ATP, we then evaluated the Ca^2+^ response to ATP in presence of CPA in PSS and in 0 [Ca^2+^]_e_ both in hCMEC/D3 cells (Fig. 7) and in iAstro-WT (Fig. 8). In the presence of extracellular Ca^2+^, CPA evoked an initial increase in [Ca^2+^]_i_ followed by a prolonged plateau phase, which were due, respectively, to passive ER Ca^2+^ release and SOCE activation (Fig. 7A-a and Fig. 8A-a). As expected, the Ca^2+^ response to CPA adopted transient kinetics under 0 [Ca^2+^]_e_ conditions (Fig. 7A-a, Fig. 7B-a). However, ATP failed to trigger intracellular Ca^2+^ signaling upon depletion of the ER Ca^2+^ store with CPA both in hCMEC/D3 cells (Fig. 7A-a and Fig. 8B-a). Furthermore, the Ca^2+^ response to ATP was abolished even when mApoE-PA-LIP was perfused upon CPA application (Fig. 7B-b; Fig. 15B-b).

## Discussion

Previous findings hinted at mApoE–PA–LIP, bifunctionalized liposomes composed of sphingomyelin (Sm) and cholesterol (Chol), as a well-tolerated valuable new nanotechnological tools for AD therapy [17], in light of their ability to bind Aβ and to target and cross the BBB [18, 32, 35]. The therapeutic effectiveness of mApoE–PA–LIP in transgenic AD mouse models has been previously reported, demonstrating their effects on brain amyloid burden reduction [17, 36] and memory improvement [17].

Starting from these evidences, we assessed mApoE-PA-LIP activities on hCMEC/D3 as an in vitro human BBB model and on cultured astrocytes in order to evaluate mApoE-PA-LIP ability of modulating the intracellular Ca^2+^ dynamics within two main cellular constituents of the NVU.

Our results proved that mApoE-PA-LIP actively modulate the intracellular Ca^2+^ waves triggered by extracellular ATP in cultured hCMEC/D3 and astrocytes.

The percentage of responding hCMEC/D3 cells increased after a pre-treatment with mApoE-PA-LIP both in standard PSS solution as well as in absence of extracellular Ca^2+^. These results could be basically related to the increased mobilization of Ca^2+^ from the intracellular stores induced by mApoE-PA-LIP mediated by the activation of the metabotropic purinergic receptors. Due to this “additional” intracellular calcium mobilization, the number of cells reaching the threshold of the ATP-evoked Ca^2+^ wave increased. Indeed, a trigger stimulus of 50 and 100 μM ATP increased the duration and the A.U.C of the Ca^2+^ wave when both hCMEC/D3 and astrocytes were pre-treated with mApoE-PA-LIP at the final concentration of 0.01mg/ml for 5 min. Interestingly, the pre-treatment with mApoE-LIP without PA functionalization failed to increase both the duration and the A.U.C of the intracellular Ca^2+^ wave triggered by ATP. mApoE-PA-LIP increased the ATP-evoked intracellular Ca^2+^ waves in cultured hCMEC/D3 and astrocytes even under 0 [Ca^2+^]_e_ conditions, thus indicating that the increased intracellular Ca^2+^ wave triggered by ATP is mainly due to endogenous Ca^2+^ release from ER. Indeed, when SERCA activity was blocked by CPA, the extracellular application of ATP failed to trigger any intracellular Ca^2+^ waves. These data are consistent with previous results by Bintig and colleagues [37], who demonstrated that the purinergic stimulation of Ca^2+^ signaling in hCMEC/D3 cells acts via G-protein-coupled P2Y2 receptor subtype that triggers Ca^2+^ wave by means of InsP3-dependent ER Ca^2+^ release.

The astrocytic Ca^2+^ signaling toolkit is remodeled after dynamic changes of astroglial morphology and function during AD [7, 9]. Therefore, it is plausible to suggest that the astrocytic response to activation of metabotropic purinergic signaling, after exposure to mApoE-PA-LIP, may depend on the state of hypo- or hyper-reactivity of astrocytes during progression of AD pathogenesis. While we show that in “healthy” cultured astrocytes, ATP-induced response was enhanced by pre-treatment with mApoE-PA-LIP, the response in astrocytes bearing FAD mutations or challenged with Aβ, both in vitro and in vivo, should be experimentally determined.

Cerebrovascular pathology is considered the major risk factor for clinically diagnosed AD-type dementia [38]. Functional changes in CBF linked with structural arterial changes are associated with the rate of accumulation of cerebral Aβ over time and the overlap of cerebrovascular and cerebral Aβ pathologies in older adults [39]. In misfolding diseases, Aβ accumulate not only in the brain but also in other organs. Therefore, the essence of AD is not purely the formation and aggregation of insoluble Aβ but rather a disorder in processes of its elimination. Here, we suggest that PA related to mApoE-PA-LIP might modulate the cell membrane curvature and promote membranes fusion, thus regulating the activity of different proteins involved in the vesicle docking. This would again indeed improve the Aβ clearance as evidenced in previous studies [17].

In addition, PA could accumulate and form microdomains highly negatively charged, which potentially serve as membrane retention sites for several key proteins for exocytosis, such as the SNARE protein syntaxin-1 [40], or other membrane remodeling processes [41]. Our mApoE-PA-LIP could at the end act as PA confined to biological membranes thus promoting the transcellular trafficking of Aβ and at the end the Aβ clearance. This evidence could indeed provide new insight to explain the “sink effect” in charge to mApoE-PA-LIP as well established by in vitro and in vivo study in mice models of AD [17, 18, 36].

Recent evidence has revealed new insights into potential role of enhanced Ca^2+^ release from ER in the context of AD [42]. Our results show that the pre-treatment with mApoE-PA-LIP, both in presence and in absence of extracellular Ca^2+^, modulates Ca^2+^ dynamics evoked by ATP when SERCA is active. In agreement with our findings related to mApoE-PA-LIP activities on intracellular Ca^2+^ waves, a recent paper by Krajnak and Dahl [43] provides evidence that agents, which actively modulate SERCA repairing Ca^2+^ unbalance, could exert neuroprotective effects and improves memory and cognition in AD model mice. Clearly, a better understanding of how dysregulation of neuronal Ca^2+^ handling contributes to neurodegeneration and neuroprotection in AD is needed as Ca^2+^ signaling modulators are targets of great interest as potential AD therapeutics [44].

Occurrence of AD symptoms is sometimes preceded by pathological changes in the brain vascular system, including accumulation of Aβ in the walls of blood vessels and lowering of CBF [45]. The activation of P2Y2 promotes the degradation of APP assisted by α-secretase, thus ending to soluble sAPPα protein rather than the neurotoxic Aβ1–42 peptide [46, 47]. Moreover, the P2Y2 receptor is important for activation of microglia cells and might affect the neuroprotective mechanisms via clearance of fibrillar Aβ1–42. Activation of P2Y2 on endothelial cells causes binding of monocytes to the endothelial wall and their diapedesis thus enhancing the neuroprotective action of the microglial cells [48]. In light of our results, we can thus speculate that the increased of the duration and A.U.C of the Ca^2+^ wave triggered by ATP when both hCMEC/D3 and iAstro-WT were pre-treated with mApoE-PA-LIP would at the end increase these neuroprotective effects.

The oxidative stress may also be counteracted via the purinergic signaling. Indeed, ADP activates P2Y13 receptors, leading to the increased activity of heme-oxygenase, which has a cytoprotective activity. Such modulating activity may be advantageous in AD [30] and mApoE-PA-LIP could indeed at the end amplify it. Purine and pyrimidine receptors are linked with the physiological function of the BBB, not only in regulating prostacyclin and NO release from the brain endothelium but also to control BBB permeability. In previous studies, it has been evidenced that mApoE-PA-LIP increased the NO synthesis and release from cultured endothelial cells [49]. The endothelium can release ATP acting on nucleotide receptors on astrocytes and neurons, being both target and source of nucleotide signals [50]. It could be of great impact that our mApoE-PA-LIP induced a positive modulation of the ATP-triggered Ca^2+^ waves both in hCMEC/D3 cells and astrocytes. Indeed, P2Y receptors, due to their subcellular expression, acting on voltage-gated membrane channels, are able to inhibit neurotransmitter release, modulate dendritic integration, facilitate neuronal excitability, or affect other various neuronal functions such as synaptic plasticity or gene expression [25]. Further studies are deserved in order to disclose the specificity of mApoE-PA-LIP in modulating neuronal synaptic transmission.

The here outlined results could give additional support to promote mApoE-PA-LIP as putative therapeutic tool for AD treatment. Indeed, targeting the neurovascular unit in AD instead of a classical neuron-centric approach in the development of neuroprotective drugs may result in improved clinical outcomes.

## Data Availability

The datasets generated during and/or analyzed during the current study are available from the corresponding author on reasonable request.
